# Perioperative Complications and Anesthesia Practices in Managing Patients With Quadriplegia Undergoing Surgery: A Systematic Review

**DOI:** 10.3389/fmed.2022.852892

**Published:** 2022-03-28

**Authors:** Abanoub Aziz Rizk, Marina Saad, Mandeep Singh, Bernhard Schaller, Lashmi Venkatraghavan, Tumul Chowdhury

**Affiliations:** ^1^Faculty of Medicine, University of Ottawa, Ottawa, ON, Canada; ^2^Department of Anesthesiology and Pain Medicine, Toronto Western Hospital, University Health Network, Toronto, ON, Canada; ^3^Department of Pathophysiology, University of Buenos Aires, Buenos Aires, Argentina

**Keywords:** quadriplegia, spinal cord injury, cardiac changes, autonomic dysreflexia, anesthesia

## Abstract

Quadriplegia is associated with a multitude of health complications affecting numerous organ systems. Complications during the perioperative periods are not uncommon in this patient population due to abnormal responses to surgical stressors. Such complications include autonomic dysreflexia, cardiac ischemia, and respiratory compromise. Currently, there is no clear consensus on the ideal technique for perioperative anesthesia management in this population. In addition, the relationship between the perioperative complications and anesthesia practices have not been explored in-depth. Therefore, we aimed to investigate perioperative complications in the context of anesthesia that are associated with patients with quadriplegia undergoing various surgical procedures. Our PRISMA compliant systematic review included 12 articles covering the literature from inception to January 12, 2021. The review showed complications being pulmonary, cerebral, but most importantly and commonly cardiac in nature, with many patients suffering hypertension, and many others hypotension. In addition, our review showed that autonomic dysreflexia is common and in majority of patients, it was managed successfully with good recovery. Based on our findings, the use of anesthesia, either general or spinal, can be considered. Future studies are needed to elucidate the exact mechanisms involved in perioperative complications and anesthetic management that are associated with patients with quadriplegia. This review will aid in developing general recommendations based on the information available in the literature to guide perioperative management of this vulnerable patient population.

## Background

Patients with quadriplegia due to cervical spinal cord injuries exhibit multiple health concerns including cardiorespiratory, gastrointestinal, cerebral neuropsychological and endocrinological dysfunctions (1–12). In addition, during the perioperative period, complications such as autonomic dysreflexia, respiratory compromise, myocardial ischemia can impose further challenges to anesthesiologists. There are few anesthetic choices for managing such patients; however, current knowledge is unclear whether there is a distinct advantage of one anesthesia technique over another (1–12). While general anesthesia is the traditional choice, some studies have indicated that a spinal anesthesia is preferred as it poses less risk of cardiac complications, namely autonomic dysreflexia (1). Unfortunately, the relationship between the various perioperative complications and anesthesia techniques were not studied extensively. Nevertheless, there is no consensus among the literature regarding a standard of care for this cohort of complex patients. Moreover, literature is limited to few case reports or observational studies (1–12), and a systematic synthesis of literature is still lacking. Therefore, it is imperative to synthesize the current understanding of the perioperative complications and outcomes pertaining to perioperative anesthesia management of quadriplegic patients undergoing various surgical procedures.

In this systematic review, we aim to investigate various perioperative complications (cardiac, cerebral, and pulmonary) that are associated with patients with quadriplegia undergoing surgical procedures. Specific objectives of the review are to evaluate the nature and extent of perioperative complications in the context of anesthesia, and the outcome of patients with quadriplegia undergoing, non-obstetric and non-spinal cord related surgeries. In addition, this synthesis will explore the various anesthetic management options for such cases to better guide perioperative patient management.

## Methods

### Population

Participants for this study comprised of all adult quadriplegic patients who had undergone non-cardiac surgeries. Patients undergoing spine and obstetric surgeries were excluded.

### Intervention and Comparator

In this review, the intervention was defined as presence of any type of anesthesia (general anesthesia, spinal anesthesia, conscious sedation, or combination). Patients with quadriplegia undergoing surgery and exhibiting similar complications without any anesthesia or only having local anesthesia were included as comparators (i.e., more than half of the total number of articles that mentioned the comparator group).

### Search Strategy

**Figure 1:**
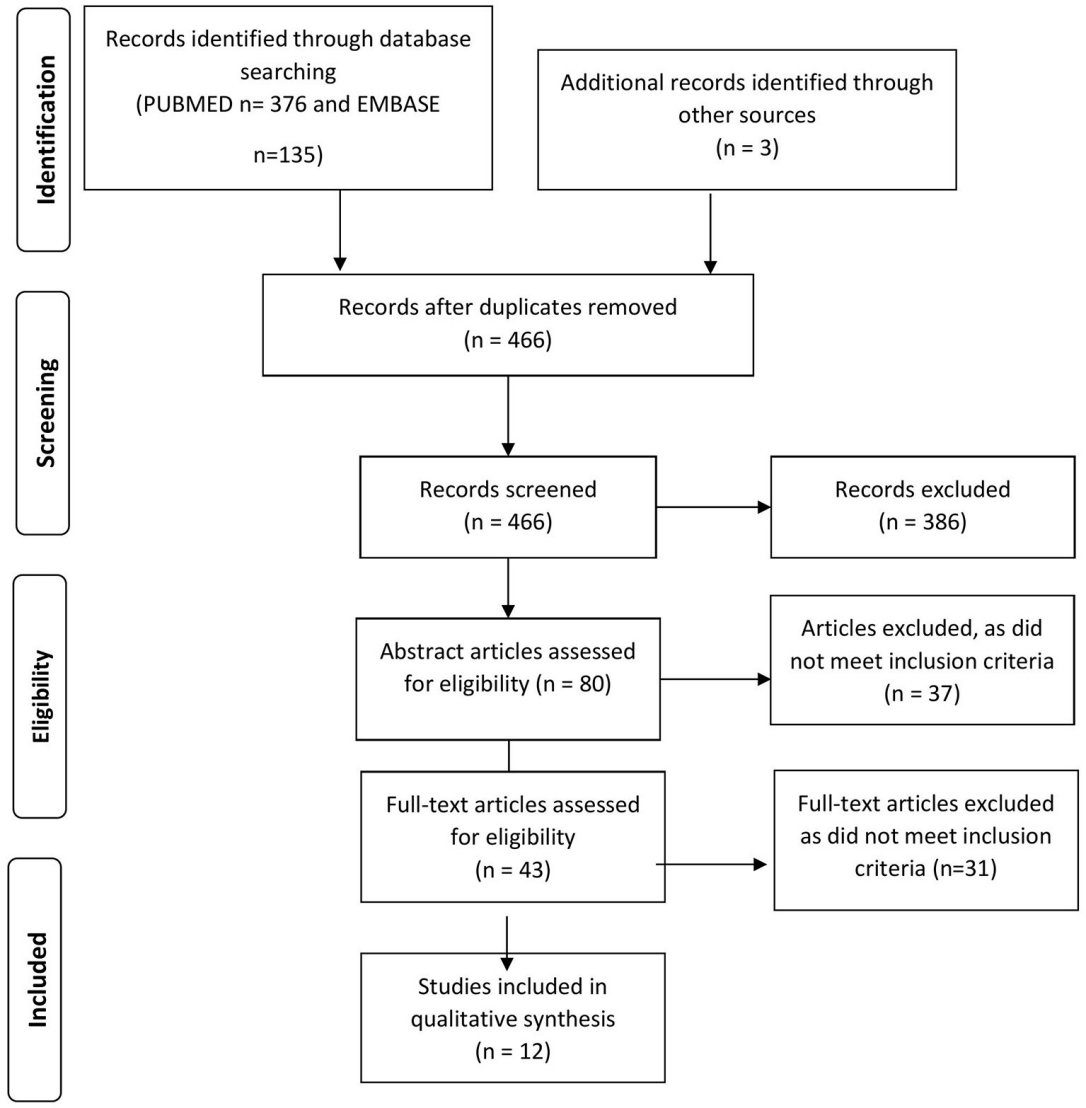
PRISMA flow diagram of search results.

The databases MEDLINE (from inception to January 12, 2021), and EMBASE (from inception to January 12, 2021), were searched. To explore the research questions this systematic review was designed following the rigorous methodology (Figure 1) (13). The operator “and” was used to combine these search results with the terms. Manual searches of reference lists and trial registries were utilized to identify additional articles. The search was restricted to the English language and human studies only. The trained researcher (AAR) developed the search strategies and is described in detail in the Supplementary Material (Supplemental Digital Content: SDC. Supplementary Table 1). The following keywords/terms were used:

(((“Quadriplegia”[Mesh]) OR ((quadripleg^*^[Title/Abstract]) OR (tetrapleg^*^[Title/Abstract]))) AND ((((((“Anesthetics”[Mesh]) OR “Anesthesia”[Mesh]) OR “Anesthesia and Analgesia”[Mesh]) OR “Hypnotics and Sedatives”[Mesh]) OR “Analgesics”[Mesh]) OR ((((anesthetic^*^[Title/Abstract]) OR (anesthes^*^[Title/Abstract])) OR (sedativ^*^[Title/Abstract])) OR (anelges^*^[Title/Abstract])))) AND (((((((((“Autonomic Dysreflexia”[Mesh]) OR “Stroke”[Mesh]) OR “Myocardial Ischemia”[Mesh]) OR “Myocardial Infarction”[Mesh]) OR “Lung Diseases”[Mesh]) OR “Respiration Disorders”[Mesh]) OR “Heart Diseases”[Mesh]) OR “Hemodynamics”[Mesh]) OR (((((((((((autonomic dysreflexia^*^[Title/Abstract]) OR (stroke^*^[Title/Abstract])) OR (myocardial infarction^*^[Title/Abstract])) OR (myocardial ischemia^*^[Title/Abstract])) OR (pulmonary deficit^*^[Title/Abstract])) OR (pulmonary dysfunction^*^[Title/Abstract])) OR (hemodynamic^*^[Title/Abstract])) OR (respiratory acidosis^*^[Title/Abstract])) OR (respiratory alkalosis^*^[Title/Abstract])) OR (pulmonary complication^*^[Title/Abstract])) OR (complication^*^[Title/Abstract]))).

### Selection Criteria and Reliability

Two independent reviewers (AAR and MS) evaluated the search results and the eligible studies for the potential inclusion were identified using the predefined selection criteria. If any disagreement occurred between the two authors, a third author (TC) was consulted to make the final decision.

### Data Extraction and Quality Assessment

All study types including randomized controlled trials, prospective and retrospective studies, case series, and case reports were screened. Based on the screening of first fifty randomized pilot (4 selected) articles, we decided to include studies based on two essential criteria: first, the article should mention at least one of the (predefined) perioperative complications and second, it should clearly describe the type of anesthesia, if any is used. There was no gender restriction for this study. We excluded studies where patients with quadriplegia had surgery during pregnancy and/or for the spine-related disorders.

All studies were graded according to the level of evidence. Methodological quality was assessed based on selection, ascertainment, causality, and reporting of observational descriptive studies (case reports/series) as mentioned by Murad et al. (14). These qualitative analyses were performed by two reviewers (MS and AAR) and bias (which included attrition bias, detection bias, and selection bias) for each study were presented as binary notion: yes and no. Moreover, non-randomized observational studies (case-control, cohort studies and quasi experimental studies) were assessed according to the Newcastle-Ottawa Scale (NOS) (15) with points being awarded based on selection, comparability, and exposure. Any discrepancy during the bias assessment was resolved with the third reviewer (LV) as to minimize selection bias. The bias assessment detail is provided in the Supplementary Material (Supplemental Digital Content: SDC. Supplementary Table 2).

### Outcome Measurements

The primary outcomes of this study were to know the incidence of three major perioperative complications (from surgery until discharge from the hospital) and their clinical presentation in patients with quadriplegia undergoing non-obstetric and non-spinal cord surgery. Major perioperative complication include: (1) ***cardiac events*** namely autonomic dysreflexia (AD) [any of the following presentation either alone or in combinations: sudden unexplained increase in systolic blood pressure (BP) > 180 mm Hg or diastolic BP > 100 mmHg, bradycardia (<60 bpm), tachycardia (>100 bpm), headache, blurred vision, severe nausea/vomiting, nasal stuffiness and sweating], myocardial ischemia/infarction [electrocardiographic (ECG) changes and or biochemical diagnosis], cardiac arrest (asystole, ventricular fibrillation), arrythmias, along with (2) ***cerebral events*** such as hemorrhagic or ischemic stroke (diagnosed on the imaging), and finally (3) ***pulmonary events*** namely apnea, oxygen desaturation requiring airway (invasive or non-invasive) intervention or change in the anesthetic technique, pulmonary embolism (confirmed with the imaging), extubation failure at the end of the surgery or continued ventilation postoperatively, and pneumonia (confirmed either radiologically or with presenting symptoms) during the hospital stay.

The secondary outcome consisted of (1) 30-day all-cause mortality and (2) overall post-surgical recovery. The latter was categorized by authors as “good” or “bad” depending on the length of hospital stay as well as unexpected medical sequelae. If studies did not mention the recovery period of patients post-operatively, it was noted as “not available.” The subgroup analysis includes delineating the type of anesthetic techniques (local anesthetics, monitored anesthesia care/sedation, regional, general anesthesia, or various combinations) and the complications.

### Data Analysis and Synthesis

Basic descriptive analysis was performed, and data was presented as means, proportions, and percentages. Data information was also presented in tabular and graphic forms, where applicable.

## Results

Our database search yielded a total of 511 articles (PubMed *n* = 376, and EMBASE *n* = 135) that were reduced to 466 results following removal of duplicates (Figure 1) and search development strategies are described in detail in the Supplementary Material (Supplemental Digital Content: SDC. Table 1). Articles were excluded in the preliminary study search based on title searches that did not meet our inclusion criteria. Next, a thorough abstract and manuscript search was conducted, and any articles that did not meet our inclusion criteria was not included. Three additional articles were searched as a cross-reference [PRISMA-P]. Following title, abstract and manuscript screens, 12 articles were finally included in the study (Tables 1–3). Nine studies were of level VI evidence, two were level IV and one was of level III (13). The articles included six case reports (50%), three case series (25%), one case control study (8%), one cohort study (8%), and one quasi-experimental study (8%).

**Table 1:** Studies and demographical characteristics.

		**Type of**	**Level of**	**No. of**		**Age**	**Cause of**	**Level of**
**No**.	**Study ID**.	**study**	**evidence**	**Patients**	**Gender**	**(years)**	**quadriplegia**	**injury**
1	Vaidyanathan et al. (1)	Case Series	VI	2	M	47, 49	Mechanical Fall MVA	C4
2	Smith et al. (2)	Case Report	VI	1	M	20	Post-operative	C4
3	Deschodt et al. (3)	Case Series	VI	7	4M;3F	22.6+/- 3.6	NR	C5–C7
4	Yoo et al. (4)	Case Report	VI	1	M	45	Mechanical Fall	C5–C6
5	Yoo et al. (5)	Case Control	IV	22	19M;3F	40+/- 13	NR	Above C7
6	Murphy et al. (6)	Case Report	VI	1	M	27	Mechanical Fall	C5
7	Raeder et al. (7)	Case Report	VI	1	M	20	MVA	C6–C7
8	Yamashita et al. (8)	Case Report	VI	1	M	37	MVA	C4–C5
9	Schonwald et al. (9)	Case Series	VI	3	3M	35	Fracture Dislocation	C5
						39	NR	Above C7
						57	NR	C7
10	Dykstra et al. (10)	Quasi-experimental	III	7	7M	21–48 (29*)	NR	C5,6 (*n* = 4),
								C4,5 (*n* = 2),
								C3,4 (*n* = 1)
11	Burnstein et al. (11)	Case Report	VI	1	M	58	Swimming Accident	C5-C6
12	Snow et al. (12)	Cohort Study	IV	35	34M;1F	32*	Various causes (e.g., Trauma, MVA)	Above C7

*M, Male; F, Female; NR, Not reported; MVA, Motor vehicular accident; N, Number*.

**Table 2:** Intra-operative characteristics.

**No**.	**Author**	**Procedure**		**Type of anesthesia**	**Anesthetic agent**	**Position of patient**
1	Vaidyanathan et al. (1)	Case 1	**Procedure A:** Cystoscopy and laser lithotripsy	Spinal Block	Bupivicaine (15 mg) IV Midazolam (1 mg)	Lithotomy
			**Procedure B:** Flexible Cystoscopy	NR	NR	Supine
			**Procedure C:** Rigid Cystoscopy and Laser lithotripsy	Sedation	IV Midazolam (3 mg)	Lithotomy
		Case 2	**Procedure A:** Laser Lithotripsy	Spinal block	Bupivacaine (17 mg)	Lithotomy
			**Procedure B:** Cystoscopy and Laser lithotripsy	No Anesthesia	N/A	Lithotomy
2	Smith et al. (2)	Dental Extraction		GA	Meperidine (75 mg) Atropine (0.4 mg) Sodium thiamylal (100 mg) d-tubocurarine (6 mg) Succinylcholine (50 mg)	Supine
3	Deschodt et al. (3)	Upper limb surgeries		Spinal Block	Bupivacaine (7.0–32.5 mg) Fentanyl (50–75 μg); (7/10) Fentanyl (25–100 μg); (6/10) Midazolam (1–2 mg); (5/10)	Lateral Decubitus/ Supine
4	Yoo et al. (4)	Debridement & primary closure of wound (post-reconstructive surgery)		LA	Lidocaine (1%)	Prone
5	Yoo et al. (5)	Different Surgical Procedures		GA	Midazolam oral (0.1 mg/kg) IV thiopental (5–7 mg/kg) IV vecuronium (0.12 mg/kg) Nitrous oxide (50%) Isoflurane in oxygen (1%)	NR
6	Murphy et al. (6)	Urinary diversion		GA	Lidocaine (2% 2 ml) Propofol (150 mg) Fentanyl (150 μg) Rocuronium (0.5 mg/kg) Nitrous oxide in oxygen (40%) Isoflurane (0.2–1.5%) Bupivacaine (10 mL, 0.25%)	Supine
7	Raeder et al. (7)	Procedure A: Removal of renal pelvic stone		Sedation	NR	Prone
		Procedure B: Removal of ureteric concerment		Sedation	NR	
8	Yamashita et al. (8)	Urethral Catheter		N/A		Supine
9	Schonwald et al. (9)	Case 1:	NR	Spinal Block	Lidocaine (75 mg)	NR
		Case 2:	Procedure A: Transurethral Sphincterotomy	Spinal Block	Tetracaine (8 mg)	Supine
		Procedure B: Penile Prosthesis		Spinal Block	Tetracaine (12 mg)	Supine
		Case 3:	Percutaneous rhizotomy	GA	Thiopental (300 mg) Succinylcholine (100 mg) Enflurane (2%)	Supine
10	Dykstra et al. (10)	Cystoscopy injection of the external urethral sphincter		N/A	NR	Supine
11	Burenstein et al. (11)	Procedure A: Cystoscopy		NR	NR	Supine
		Procedure B: Extra shock wave Lithotripsy		Sedation	Midazolam (1.5 mg) Midazolam (0.5 mg)	
		Procedure C: Cystoscopy and Extra shock wave lithotripsy		NR	NR	
12	Snow et al. (12)	Cystoscopy		GA + Sedatives	Diazepam (10 mg) Pentobarbital (100 mg) Thiopental (25–50 mg) Nitrous Oxide, Oxygen	Various depending on procedure

*NR, Not reported; N/A, Not applicable; GA, General Anesthetic; LA, Local Anesthetic*.

**Table 3:** Study outcomes.

**No**.	**Study ID**	**Type of complication**		**Complication specifics**	**Management**	**Outcomer**
1	Vaidyanathan et al. (1)	Case 1	P1: None	N/A	N/A	N/A
			P2: None	N/A	N/A	N/A
			P3: Cardiac	Hypertension	Tramadol IVx3 (25 mg) Labetalol (5 mg) Propofol (120 mg)	Severe bleeding from hyperaemic bladder mucosa Surgery abandoned.
		Case 2	P1: None	N/A	N/A	N/A
			P2: Cardiac	Hypertension Bradycardia Headache	IV Midazolam (2 mg) IV Glycopyrrolate (50 μg) IV Labetalol (10 mg)	Uneventful recovery
2	Smith et al. (2)	Cardiac (1)		Cardiac arrest Ventricular fibrillation.	Ventilation External cardiac compression Defibrillation	Defibrillated successfully on third attempt. Extubated uneventfully Uneventful recovery
3	Deschodt et al. (3)	Cardiac Pulmonary Cerebral		Painful paresthesia (*n* = 1) Apnea (*n* = 1) Bradycardia (*n* = 5)	Face mask ventilation IV atropine (*n* = 1) (0.25 mg)	Pulmonary function restored. Uneventful recovery
4	Yoo et al. (4)	Cardiac Cerebral		Headache Abnormal mental status Diaphoresis Hypertension (268/185 mmHg) Bradycardia Confusion Numbness Coma (Glascow Coma Scale Score = 3) Intracranial hemorrhage	Prone to supine position T1reated for the possible precipitating factors of AHR. Unblocked Foley Catheter IV Hydralazine (10 mg) Nitroglycerin External Ventricular Drains Mannitol Transferred to ICU Mechanical Ventilation	Surgery immediately stopped. Hemodynamic and neurological status deteriorated. Died nine days after episode.
5	Yoo et al. (5)	Cardiac		Hypertension: QP (*n* = 2), control (*n* = 10) Hypotension: QP (*n* = 9), control (*n* = 1) Tachycardia: QP (*n* = 1), control (*n* = 3) Bradycardia: QP (*n* = 1), control (*n* = 0) Dysrhythmia: QP (*n* = 1), Control (*n* = 2)	NR	Arrythmias disappeared spontaneously without treatment
6	Murphy et al. (6)	Cardiac		Autonomic Dysreflexia^+^	Epidural top-up	Complete resolution after 12 min Uneventful recovery
7	Raeder et al. (7)	Cardiac		Hypertension (230/140 mmHg)	Deepening of anesthesia Increments of Fentanyl	Uneventful recovery
		Cardiac Cerebral		Hypertension (220/130 mmHg) Tachycardia (120 bpm) Bladder spasm^+^ Headache^+^ Profuse sweating^+^ Tachycardia^+^ Blurred vision^+^ Total Circulatory collapse + Convulsions^+^ Bradycardia^+^ Unconsciousness^+^ Apnea^+^	**Intraoperatively** Deepening anesthesia **Post-op (2 days)** Ventilated IV Propranolol (Tachycardia) Atropine (Bradycardia) Emepronium bromide IM x3 (200mg) Emepronium bromide IM x3 (50mg)	**Intraoperatively** Symptoms relieved **Post-op (2 days)** Uneventful recovery
8	Yamashita et al. (8)	Cardiac Cerebral		Bradycardia Multiple subcortical and intracranial hemorrhage	Intraoperatively Nicardipine *Post-*op Anticonvulsants Intubation + mechanical ventilation	Patient died 3 weeks after intracranial hemorrhage.
9	Schonwald et al. (9)	Case 1	Cardiac Pulmonary Cerebral	Shortness of Breath Facial tingling Nausea Hypertension (220) Bradycardia (30 bpm)	Cystoscope removed. Bladder emptied. General Anesthesia induced (2.5% halothane)	Symptoms relieved Uneventful recovery
		Case 2	P1: Cardiac	Bradycardia Asystole	IV atropine (0.4mg) IV fluids Placed in Trendelenburg position	Uneventful recovery
			P2: Cardiac	Bradycardia (50bpm) Hypotension (90)	No treatment	Uneventful recovery
		Case 3	Cardiac	Hypotension (50) when prone	IV fluids Ephedrine (10 mg) Decreasing depth of anesthesia	Uneventful recovery
10	Dykstra et al. (10)	Cerebral Cardiac		Headache Blurred vision Sweating Flushing Hypertension	Sublingual Nifedipine	Symptoms relieved Uneventful recovery
11	Burenstein et al. (11)	Procedure A: Cardiac		Autonomic Dysreflexia	No medication given	Surgery abandoned
		Procedure B: Cardiac Cerebral		Hypertension (240/123) Head discomfort	10 mg sublingual nifedipine	Surgery abandoned.
		Procedure C: Cardiac		Pre-op Hypertension (150/80) Hypotension (80/60) Bradycardia (40 bpm)	Pre-op Sublingual Nifedipine (prophylactically) (10 mg) Ephedrine (5 mg) Atropine (0.4 mg)	Surgery and recovery uneventful
12	Snow et al. (12)	Cardiac		Hypertension (28/35 pts) Bradycardia	Trimethaphan 0.1%, (10–50 mg)	Mild allergic reaction to Trimethaphan. (*n* = 2) Uneventful recoveries

*NR, Not reported; N/A, Not applicable; P, Procedure; “+” superscript signifying post-operative complication vs. no superscript signifying intra-operative complication*.

*Demographic characteristics*: The studies included a total of 82 patients with 75 males (91%) and 7 female (9%) with an average age of 37 years (range 20-58 years) (Table 1). More than half the patients had quadriplegia due to the previous trauma from mechanical falls or accidents (54%) while the remaining group had quadriplegia due to an unspecified cause (46%). Majority (60%) of patients underwent urological procedures.

### Primary Outcome

In our study, majority of perioperative complications were reported during intraoperative phase (10, *n* = 12 articles) and only (2, *n* = 12 articles) reported during the postoperative period. Regarding the complications, all 82 patients had cardiac events (100%) with the majority (54%) experiencing autonomic dysreflexia (presenting as hypertension (44 out of 82) and bradycardia (13 out of 82) followed by hypotension (11 out of 82), and tachycardia (2 out of 82). Cerebral and pulmonary events were reported in 7 (8.5%) and 2 (2.5%) patients, respectively.

Management of complications varied with the type of surgery and the severity of the complication. In 95% of patients, intraoperative complications were successfully managed medically without the cancellation of the surgery or procedure. However, in the remaining 5% of patients with severe complications, the surgery was terminated. In all terminated surgeries, patients had severe hypertension which met criteria for autonomic dysreflexia.

### Other Outcomes and Subgroup Analysis

The 82 patients included in the study underwent a total of 89 procedures. General anesthesia was the most commonly (68.5%) used anesthesia technique followed by the spinal anesthesia (13.5%). Three procedures were done under conscious sedation, one procedure was done under local anesthesia alone while nine procedures did not require general anesthesia. (Table 4) Both sedation and spinal block anesthesia led to cardiac (bradycardia, hypotension, asystole, hypertension, sweating), pulmonary (shortness of breath, apnea), and cerebral complications (facial tingling, painful paresthesia, headaches, blurred vision, convulsions). On the other hand, local anesthesia reported only cardiac (sweating, hypertension) and cerebral complications (headaches, abnormal mental status, confusion, numbness, coma, and intracranial hemorrhage). In addition, general anesthesia protocols lead to only cardiac complications (cardiac arrest, ventricular fibrillation, hypertension, hypotension, tachycardia, bradycardia, dysrhythmia, autonomic dysreflexia). Lastly, not utilizing any anesthesia still caused cardiac (autonomic dysreflexia, hypotension, hypertension, bradycardia) and cerebral complications (headache, intracranial hemorrhage, blurred vision). While 10 out of the 12 articles did not comment on the 30-day mortality, only two deaths were reported following the procedures (9 and 13 days postoperatively, respectively). The length of hospital stay ranged from 1 day to 1 month, however the remaining 10 articles of studies did not report any follow-up periods.

**Table 4:** Complications organized by categories: cardiac, pulmonary, cerebral and others.

	**Cardiac complications (# of studies reported)**	**Pulmonary complications (# of studies reported)**	**Cerebral complications (# of studies reported)**	**Other complications (# of studies reported)**
Spinal block	Bradycardia Hypotension Asystole Hypertension	Shortness of Breath Apnea	Facial Tingling Painful Paresthesia	Nausea
Sedation	Hypertension Bradycardia Tachycardia Sweating	Apnea	Headaches Blurred Vision Convulsions	Bladder spasms
Local anesthesia	Sweating Hypertension		Headaches Abnormal Mental Status Confusion Numbness Coma Intracranial hemorrhage	
General anesthesia	Cardiac Arrest Ventricular Fibrillation Hypertension Hypotension Tachycardia Bradycardia Dysrhythmia Autonomic dysreflexia			
No Anesthesia	Autonomic Dysreflexia Hypotension Hypertension Bradycardia		Headache Intracranial Hemorrhage Blurred vision	Sweating Flushing

*Exact number of cases per symptom could not be identified as majority of articles did not include this information. Empty boxes refer to no reported complications within that category*.

## Discussion

Our systematic review highlights some interesting clinical findings pertaining elements to major perioperative complications and anesthetic choices in patients with quadriplegia undergoing non-obstetric and non-spine related procedures/surgeries.

### Perioperative Complications

With regards to the primary outcome, most complications were cardiac in nature, and interestingly many patients experienced hypertension, and others experienced hypotension in this review. This is in line with current literature where important perioperative complications reported include autonomic dysreflexia, bradycardia, hypotension, respiratory inadequacy, and muscle spasms (16).

**Figure 2:**
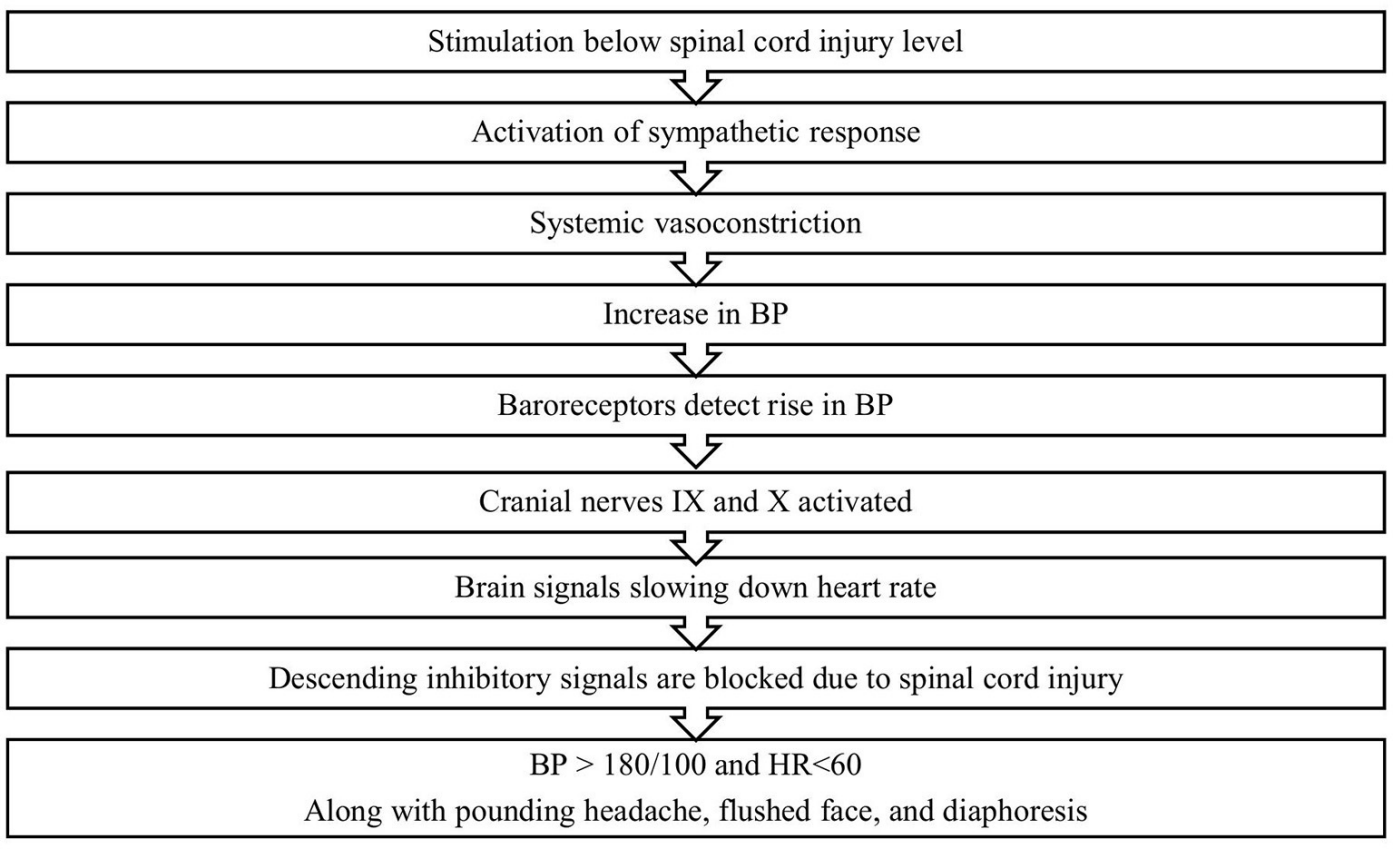
Mechanisms of autonomic dysreflexia.

Autonomic Dysreflexia (AD) is a condition arising from previous spinal cord injury with severity and chance increasing as the level of injury increases (Figure 2) (17). AD is a life-threatening condition in which autonomic responses are dysregulated leading to severe hypertension in response to a noxious stimulus from below the site of injury (17). In about 85% of cases, this stimulus is caused by a urological procedure or issue such as a distended bladder, a problematic Foley catheter or an infection (17, 18). This is consistent with our findings, as the majority of our patients underwent a urological procedure. In addition, while patients with AD are at a much higher risk of death, our study only presented one patient who died within 30 days of the procedure (17). AD can also lead to severe cardiovascular dysfunction and painful headaches but objectively it is measured as a sudden increase in systolic blood pressure by 25 mm Hg (17). In addition, in line with our findings, AD occurs more frequently during urological surgery than any other surgery (17–19). Therefore, prior to surgery, triggers must be identified such as checking for catheter blockages, ensuring empty bowels and attention to skin changes or ulcerations (20). It is also recorded that some urological conditions may precede AD (21, 22).

Pulmonary and cerebral complications as part of the above-described AD were also observed during the procedures. While in some patients' vital capacities and ventilations are spared, their vital capacity and ventilation are reduced due to intercostal paralysis (3). As a consequence, patient must be given low concentrations of local anesthetics, and should be informed that if complications occur it might be necessary to switch to general anesthesia (3). In addition, a study showed a patient suffering from cerebral complications such as intracranial hemorrhage, headaches, irritability, and diaphoresis in response to AD (4). High catecholamine release in response to a noxious stimulus may be an explanation to the hypertensive crisis seen in these patients (4). Transformation of the anesthetic management should therefore be considered in such patients.

### Choice of Anesthesia

In perioperative setting, patients with complete upper spinal cord injury have three options for anesthesia: monitored anesthesia care (with or without conscious sedation), regional anesthesia, and general anesthesia (20). Some patients may not require anesthetic agents if their surgery is below the site of injury, however, these patients must be well-educated on the risks and symptoms of AD and be asked to immediately report any changes such as headache, sweating, or difficulty breathing (23). Additional caution must be used when utilizing sedative agents as it may mask some symptoms due to ventilatory depression (20). In addition, patients may also choose to receive regional anesthesia. Spinal anesthesia appears to be a safer option and has become more and more widely accepted as it reduces risks of AD, and of spasms (20). Unfortunately, effectiveness and level of block have been reported to be difficult to ascertain, and therefore careful watching is required. Premedication using nifedipine has been shown to be effective, however anticholinergics have not shown much benefit unless it induces detrusor areflexia (20).

In our review, majority of studies have utilized some form of anesthesia technique, which is in line with the literature suggesting that perioperative complications are more common in patients who do not use anesthetic agents (16). Although there are few articles discussing the safety of not using any anesthetic agents, the ones present recommended that lack of anesthesia should be considered only with extreme caution and with an anesthesiologist on standby (23). This is supported by our review as two patients declined both general and spinal anesthesia during their cystoscopy procedures. Severe cardiac complications were reported in two cases where no anesthetic technique was utilized. In one patient, spinal anesthesia was offered that was associated with an uneventful postoperative period. It is prudent to block the afferent pathway of the AD by utilizing local, or regional anesthesia technique (1, 4). Therefore, our systematic review suggests that use of either general or spinal anesthesia is recommended over the sedation or local anesthesia.

Both general and spinal anesthesia are commonly used in surgical procedures for patients with high spinal cord injury. As of now, there is no consensus in the literature regarding a standard of care that should be applied for all patients.

### Management of Complications

Our review showed that the AD is common and in majority of patients it was managed successfully with good recovery. This finding is in line with the literature, in which agents such as nifedipine, nitrates and captopril were preventively utilized with success (20). In some centers for example, 10 mg of oral nifedipine is given an hour prior to surgery to prevent AD (16). Although pharmacologic methods seem effective, identifying and eliminating triggers of AD, such as a distended bladder, have been utilized as first line treatment based on expert opinion and physiology rationale (17, 18).

### Recommendations

Our review has attempted to provide better understanding of the AD after quadriplegia. A range of afferent stimuli can trigger an AD reaching the isolated spinal cord caudal to the level of spinal cord damage, mostly of visceral but less commonly of cutaneous and proprioceptive nature (17). This trigger can persist through the whole perioperative period including the immediate postoperative period. This systematic review emerged to shed some insights to both clinical and neurophysiological findings that could aid healthcare teams to better take care of this cohort of patients. Based on the available literature and expert opinion, it is reasonable to recommend following a few steps:

Patients with high spinal lesions without severe respiratory compromise undergoing non-obstetric and non-spinal surgery appear to be at an increased risk of developing perioperative complications, especially cardiac. Therefore, in such a context, risks and benefits of using particular anesthetic agents should be incorporated into the discussion. The literature recommends spinal blocks as a safe and effective method for these patients, but most procedures in our review utilize general anesthesia and perioperative complications were adequately managed (2, 5, 6, 11, 12). In addition, even for minor surgeries, one should be cautious in using local anesthesia with or without sedation (if the site of surgery is below the injury level) and patients should be fully aware of potential risks such as headache, sweating, or palpitation, difficulty breathing etc. The Presence of anesthesiologist for such cases is warranted. Agents, such as Nifedipine, should also be readily accessible to minimize or even prevent the effects of these complications. Patient reassurance can be provided given most patients had uneventful recoveries post-administration of a reversal agent or by deepening of the anesthetic agent, which is in line with our findings. In summary, general or spinal anesthesia is recommended over the sedation or local anesthesia.

### Limitations

There are several limitations to our systematic review. Overall, there was a limited number of included studies looking at perioperative complications in patients with quadriplegia undergoing non-labor and non-spinal cord related surgeries (*n* = 12), but our sensitivity analysis showed similar results. In addition, most of the included articles were of evidence level IV or higher. As a result, the overall conclusions regarding this cohort of patients undergoing surgery and their management are limited. However, our strict methodology in the study design of this review, has led to upgraded or double upgraded according to the GRADE system, (24) so that we have moderate to high quality of included studies, what gives sufficient evidence for our findings. Nevertheless, the results of this review should be considered preliminary with the aim to motivate future prospective studies.

Also, both selection and attrition bias may be present in our study given the low number of articles and the heterogeneity in study data. On the other hand, all the data was organized in a standardized way, and many articles showed similar outcomes despite such differences, which is reflected in our sensitivity analysis, so that our results have good evidence.

Another limitation of our study involves the low number of female patients. This is backed by studies showing that males are in general more likely to suffer quadriplegia compared to females. Due to this, conclusions may be biased toward male patients, and more data would be needed to gain greater generalizability.

## Conclusions

This systematic reviewshows various operative and anesthetic considerations for patients with quadriplegia undergoing various surgical procedures. The study highlighted intra-operative complications including those of cardiac, cerebral, and pulmonary nature as well as management strategies, overall patient outcomes, and anesthetic agents used. The work shows that a detailed anesthetic planning related to neurological but also neurophysiological details of each individual case is necessary to choose the best anesthetic approach.

## Data Availability Statement

The raw data supporting the conclusions of this article will be made available by the authors, without undue reservation.

## Author Contributions

AR and MSa have contributed substantially for data search, synthesis and writing. MSi, BS, and LV have contributed in writing, editing and revising the manuscript. TC has contributed substantially on the concept development, data synthesis, interpretation, writing, and editing the manuscript. All authors contributed to the article and approved the submitted version.

## Funding

MSi was supported by the Canadian Anesthesiology Society Career Scientist Award, and the merit awards program, department of anesthesiology and pain medicine, University of Toronto.

## Conflict of Interest

The authors declare that the research was conducted in the absence of any commercial or financial relationships that could be construed as a potential conflict of interest.

## Publisher's Note

All claims expressed in this article are solely those of the authors and do not necessarily represent those of their affiliated organizations, or those of the publisher, the editors and the reviewers. Any product that may be evaluated in this article, or claim that may be made by its manufacturer, is not guaranteed or endorsed by the publisher.
