# Supplementary material for: Perioperative Complications and Anesthesia Practices in Managing Patients With Quadriplegia Undergoing Surgery: A Systematic Review
Source: Front Med (Lausanne). 2022 Mar 28;9:852892. doi: 10.3389/fmed.2022.852892 (PMC8996073; doi:10.3389/fmed.2022.852892)
Supplement: Supplementary file 1 [file Table_1.DOCX]

Supplemental Digital Content: SDC. Table 1: Quality assessment of studies

1. Nonrandomized Studies ^15^

|  | Selection | Comparability | Exposure |
| --- | --- | --- | --- |
| Yoo et al., 2001 |  |  |  |
| Snow et al., 1977 |  |  |  |

1. Case series and reports ^14^

|  | **Selection** | **Ascertainment** | | **Causality** | | | | **Reporting** |
| --- | --- | --- | --- | --- | --- | --- | --- | --- |
|  | Does the patient(s) represent(s) the whole experience of the investigator (centre)? (or is the selection method unclear to the extent that other patients with similar presentation may not have been reported?) | Was the exposure adequately ascertained? | Was the outcome adequately ascertained? | Were other alternative causes that may explain the observation ruled out? | Was there a challenge/rechallenge phenomenon? | Was there a dose–response effect? | Was follow-up long enough for outcomes to occur? | Is the case(s) described with sufficient details to allow other investigators to replicate the research or to allow practitioners make inferences related to their own practice? |
| S. Vaidyanathan et al., 2012) | Y | Y | Y | N | N | N | Y | Y |
| Smith et al., 1970 | Y | Y | Y | N | N | N | Y | Y |
| Deschodt et al., 2002^4^ | Y | Y | Y | N | N | N | Y | Y |
| Yoo et al.,2010^5^ | Y | Y | Y | Y | N | N | Y | Y |
| Murphy et al., 1999^8^ | Y | Y | Y | Y | N | N | Y | Y |
| Raeder et al., 1986 | Y | N | Y | N | N | N | Y | Y |
| Yamashita et al., 2011 | Y | N | Y | Y | N | N | Y | Y |
| Schonwald et al., 1981 | Y | Y | Y | N | N | N | Y | Y |
| Dykstra, et al., 1987 | Y | Y | Y | Y | N | N | Y | Y |
| Burnstein et al., 1991 | Y | N | Y | N | N | N | Y | Y |

Y = Yes (1 Point)

N = No (0 Points)
