# Supplementary material for: Perioperative Complications and Anesthesia Practices in Managing Patients With Quadriplegia Undergoing Surgery: A Systematic Review
Source: Front Med (Lausanne). 2022 Mar 28;9:852892. doi: 10.3389/fmed.2022.852892 (PMC8996073; doi:10.3389/fmed.2022.852892)
Supplement: Supplementary file 2 [file Table_2.DOCX]

Supplemental Digital Content: SDC. Table 2: Detailed search strategies

**PUBMED**

Bottom of Form

## History and Search Details

Top of Form

Download

Bottom of Form

Delete

| **Search** | **Actions** | **Details** | **Query** | **Results** | **Time** |
| --- | --- | --- | --- | --- | --- |
| #13 |  |  | Search: **((("Quadriplegia"[Mesh]) OR ((quadripleg*[Title/Abstract]) OR (tetrapleg*[Title/Abstract]))) AND (((((("Anesthetics"[Mesh]) OR "Anesthesia"[Mesh]) OR "Anesthesia and Analgesia"[Mesh]) OR "Hypnotics and Sedatives"[Mesh]) OR "Analgesics"[Mesh]) OR ((((anesthetic*[Title/Abstract]) OR (anesthes*[Title/Abstract])) OR (sedativ*[Title/Abstract])) OR (anelges*[Title/Abstract])))) AND ((((((((("Autonomic Dysreflexia"[Mesh]) OR "Stroke"[Mesh]) OR "Myocardial Ischemia"[Mesh]) OR "Myocardial Infarction"[Mesh]) OR "Lung Diseases"[Mesh]) OR "Respiration Disorders"[Mesh]) OR "Heart Diseases"[Mesh]) OR "Hemodynamics"[Mesh]) OR (((((((((((autonomic dysreflexia*[Title/Abstract]) OR (stroke*[Title/Abstract])) OR (myocardial infarction*[Title/Abstract])) OR (myocardial ischemia*[Title/Abstract])) OR (pulmonary deficit*[Title/Abstract])) OR (pulmonary dysfunction*[Title/Abstract])) OR (hemodynamic*[Title/Abstract])) OR (respiratory acidosis*[Title/Abstract])) OR (respiratory alkalosis*[Title/Abstract])) OR (pulmonary complication*[Title/Abstract])) OR (complication*[Title/Abstract])))** | [135](https://pubmed.ncbi.nlm.nih.gov/?term=%28%28%28%22Quadriplegia%22%5BMesh%5D%29+OR+%28%28quadripleg%2A%5BTitle%2FAbstract%5D%29+OR+%28tetrapleg%2A%5BTitle%2FAbstract%5D%29%29%29+AND+%28%28%28%28%28%28%22Anesthetics%22%5BMesh%5D%29+OR+%22Anesthesia%22%5BMesh%5D%29+OR+%22Anesthesia+and+Analgesia%22%5BMesh%5D%29+OR+%22Hypnotics+and+Sedatives%22%5BMesh%5D%29+OR+%22Analgesics%22%5BMesh%5D%29+OR+%28%28%28%28anesthetic%2A%5BTitle%2FAbstract%5D%29+OR+%28anesthes%2A%5BTitle%2FAbstract%5D%29%29+OR+%28sedativ%2A%5BTitle%2FAbstract%5D%29%29+OR+%28anelges%2A%5BTitle%2FAbstract%5D%29%29%29%29+AND+%28%28%28%28%28%28%28%28%28%22Autonomic+Dysreflexia%22%5BMesh%5D%29+OR+%22Stroke%22%5BMesh%5D%29+OR+%22Myocardial+Ischemia%22%5BMesh%5D%29+OR+%22Myocardial+Infarction%22%5BMesh%5D%29+OR+%22Lung+Diseases%22%5BMesh%5D%29+OR+%22Respiration+Disorders%22%5BMesh%5D%29+OR+%22Heart+Diseases%22%5BMesh%5D%29+OR+%22Hemodynamics%22%5BMesh%5D%29+OR+%28%28%28%28%28%28%28%28%28%28%28autonomic+dysreflexia%2A%5BTitle%2FAbstract%5D%29+OR+%28stroke%2A%5BTitle%2FAbstract%5D%29%29+OR+%28myocardial+infarction%2A%5BTitle%2FAbstract%5D%29%29+OR+%28myocardial+ischemia%2A%5BTitle%2FAbstract%5D%29%29+OR+%28pulmonary+deficit%2A%5BTitle%2FAbstract%5D%29%29+OR+%28pulmonary+dysfunction%2A%5BTitle%2FAbstract%5D%29%29+OR+%28hemodynamic%2A%5BTitle%2FAbstract%5D%29%29+OR+%28respiratory+acidosis%2A%5BTitle%2FAbstract%5D%29%29+OR+%28respiratory+alkalosis%2A%5BTitle%2FAbstract%5D%29%29+OR+%28pulmonary+complication%2A%5BTitle%2FAbstract%5D%29%29+OR+%28complication%2A%5BTitle%2FAbstract%5D%29%29%29&ac=no&sort=relevance) | 15:43:14 |
| #12 |  |  | Search: **("Quadriplegia"[Mesh]) OR ((quadripleg*[Title/Abstract]) OR (tetrapleg*[Title/Abstract]))** | [12,496](https://pubmed.ncbi.nlm.nih.gov/?term=%28%22Quadriplegia%22%5BMesh%5D%29+OR+%28%28quadripleg%2A%5BTitle%2FAbstract%5D%29+OR+%28tetrapleg%2A%5BTitle%2FAbstract%5D%29%29&ac=no&sort=relevance) | 15:42:56 |
| #11 |  |  | Search: **((((("Anesthetics"[Mesh]) OR "Anesthesia"[Mesh]) OR "Anesthesia and Analgesia"[Mesh]) OR "Hypnotics and Sedatives"[Mesh]) OR "Analgesics"[Mesh]) OR ((((anesthetic*[Title/Abstract]) OR (anesthes*[Title/Abstract])) OR (sedativ*[Title/Abstract])) OR (anelges*[Title/Abstract]))** | [586,233](https://pubmed.ncbi.nlm.nih.gov/?term=%28%28%28%28%28%22Anesthetics%22%5BMesh%5D%29+OR+%22Anesthesia%22%5BMesh%5D%29+OR+%22Anesthesia+and+Analgesia%22%5BMesh%5D%29+OR+%22Hypnotics+and+Sedatives%22%5BMesh%5D%29+OR+%22Analgesics%22%5BMesh%5D%29+OR+%28%28%28%28anesthetic%2A%5BTitle%2FAbstract%5D%29+OR+%28anesthes%2A%5BTitle%2FAbstract%5D%29%29+OR+%28sedativ%2A%5BTitle%2FAbstract%5D%29%29+OR+%28anelges%2A%5BTitle%2FAbstract%5D%29%29&ac=no&sort=relevance) | 15:42:36 |
| #10 |  |  | Search: **(((((((("Autonomic Dysreflexia"[Mesh]) OR "Stroke"[Mesh]) OR "Myocardial Ischemia"[Mesh]) OR "Myocardial Infarction"[Mesh]) OR "Lung Diseases"[Mesh]) OR "Respiration Disorders"[Mesh]) OR "Heart Diseases"[Mesh]) OR "Hemodynamics"[Mesh]) OR (((((((((((autonomic dysreflexia*[Title/Abstract]) OR (stroke*[Title/Abstract])) OR (myocardial infarction*[Title/Abstract])) OR (myocardial ischemia*[Title/Abstract])) OR (pulmonary deficit*[Title/Abstract])) OR (pulmonary dysfunction*[Title/Abstract])) OR (hemodynamic*[Title/Abstract])) OR (respiratory acidosis*[Title/Abstract])) OR (respiratory alkalosis*[Title/Abstract])) OR (pulmonary complication*[Title/Abstract])) OR (complication*[Title/Abstract]))** | [3,772,187](https://pubmed.ncbi.nlm.nih.gov/?term=%28%28%28%28%28%28%28%28%22Autonomic+Dysreflexia%22%5BMesh%5D%29+OR+%22Stroke%22%5BMesh%5D%29+OR+%22Myocardial+Ischemia%22%5BMesh%5D%29+OR+%22Myocardial+Infarction%22%5BMesh%5D%29+OR+%22Lung+Diseases%22%5BMesh%5D%29+OR+%22Respiration+Disorders%22%5BMesh%5D%29+OR+%22Heart+Diseases%22%5BMesh%5D%29+OR+%22Hemodynamics%22%5BMesh%5D%29+OR+%28%28%28%28%28%28%28%28%28%28%28autonomic+dysreflexia%2A%5BTitle%2FAbstract%5D%29+OR+%28stroke%2A%5BTitle%2FAbstract%5D%29%29+OR+%28myocardial+infarction%2A%5BTitle%2FAbstract%5D%29%29+OR+%28myocardial+ischemia%2A%5BTitle%2FAbstract%5D%29%29+OR+%28pulmonary+deficit%2A%5BTitle%2FAbstract%5D%29%29+OR+%28pulmonary+dysfunction%2A%5BTitle%2FAbstract%5D%29%29+OR+%28hemodynamic%2A%5BTitle%2FAbstract%5D%29%29+OR+%28respiratory+acidosis%2A%5BTitle%2FAbstract%5D%29%29+OR+%28respiratory+alkalosis%2A%5BTitle%2FAbstract%5D%29%29+OR+%28pulmonary+complication%2A%5BTitle%2FAbstract%5D%29%29+OR+%28complication%2A%5BTitle%2FAbstract%5D%29%29&ac=no&sort=relevance) | 15:42:23 |
| #9 |  |  | Search: **(quadripleg*[Title/Abstract]) OR (tetrapleg*[Title/Abstract])** | [8,592](https://pubmed.ncbi.nlm.nih.gov/?term=%28quadripleg%2A%5BTitle%2FAbstract%5D%29+OR+%28tetrapleg%2A%5BTitle%2FAbstract%5D%29&ac=no&sort=relevance) | 15:42:04 |
| #8 |  |  | Search: **(((anesthetic*[Title/Abstract]) OR (anesthes*[Title/Abstract])) OR (sedativ*[Title/Abstract])) OR (anelges*[Title/Abstract])** | [251,456](https://pubmed.ncbi.nlm.nih.gov/?term=%28%28%28anesthetic%2A%5BTitle%2FAbstract%5D%29+OR+%28anesthes%2A%5BTitle%2FAbstract%5D%29%29+OR+%28sedativ%2A%5BTitle%2FAbstract%5D%29%29+OR+%28anelges%2A%5BTitle%2FAbstract%5D%29&ac=no&sort=relevance) | 15:40:19 |
| #7 |  |  | Search: **((((((((((autonomic dysreflexia*[Title/Abstract]) OR (stroke*[Title/Abstract])) OR (myocardial infarction*[Title/Abstract])) OR (myocardial ischemia*[Title/Abstract])) OR (pulmonary deficit*[Title/Abstract])) OR (pulmonary dysfunction*[Title/Abstract])) OR (hemodynamic*[Title/Abstract])) OR (respiratory acidosis*[Title/Abstract])) OR (respiratory alkalosis*[Title/Abstract])) OR (pulmonary complication*[Title/Abstract])) OR (complication*[Title/Abstract])** | [1,539,422](https://pubmed.ncbi.nlm.nih.gov/?term=%28%28%28%28%28%28%28%28%28%28autonomic+dysreflexia%2A%5BTitle%2FAbstract%5D%29+OR+%28stroke%2A%5BTitle%2FAbstract%5D%29%29+OR+%28myocardial+infarction%2A%5BTitle%2FAbstract%5D%29%29+OR+%28myocardial+ischemia%2A%5BTitle%2FAbstract%5D%29%29+OR+%28pulmonary+deficit%2A%5BTitle%2FAbstract%5D%29%29+OR+%28pulmonary+dysfunction%2A%5BTitle%2FAbstract%5D%29%29+OR+%28hemodynamic%2A%5BTitle%2FAbstract%5D%29%29+OR+%28respiratory+acidosis%2A%5BTitle%2FAbstract%5D%29%29+OR+%28respiratory+alkalosis%2A%5BTitle%2FAbstract%5D%29%29+OR+%28pulmonary+complication%2A%5BTitle%2FAbstract%5D%29%29+OR+%28complication%2A%5BTitle%2FAbstract%5D%29&ac=no&sort=relevance) | 15:38:39 |
| #5 |  |  | Search: **"Quadriplegia"[Mesh]** Sort by: **Most Recent** | [8,100](https://pubmed.ncbi.nlm.nih.gov/?sort=date&term=%22Quadriplegia%22%5BMesh%5D) | 15:34:28 |
| #3 |  |  | Search: **(((("Anesthetics"[Mesh]) OR "Anesthesia"[Mesh]) OR "Anesthesia and Analgesia"[Mesh]) OR "Hypnotics and Sedatives"[Mesh]) OR "Analgesics"[Mesh]** Sort by: **Most Recent** | [471,030](https://pubmed.ncbi.nlm.nih.gov/?sort=date&term=%28%28%28%28%22Anesthetics%22%5BMesh%5D%29+OR+%22Anesthesia%22%5BMesh%5D%29+OR+%22Anesthesia+and+Analgesia%22%5BMesh%5D%29+OR+%22Hypnotics+and+Sedatives%22%5BMesh%5D%29+OR+%22Analgesics%22%5BMesh%5D) | 15:31:22 |
| #1 |  |  | Search: **((((((("Autonomic Dysreflexia"[Mesh]) OR "Stroke"[Mesh]) OR "Manestheticyocardial Ischemia"[Mesh]) OR "Myocardial Infarction"[Mesh]) OR "Lung Diseases"[Mesh]) OR "Respiration Disorders"[Mesh]) OR "Heart Diseases"[Mesh]) OR "Hemodynamics"[Mesh]** Sort by: **Most Recent** | [2,746,088](https://pubmed.ncbi.nlm.nih.gov/?sort=date&term=%28%28%28%28%28%28%28%22Autonomic+Dysreflexia%22%5BMesh%5D%29+OR+%22Stroke%22%5BMesh%5D%29+OR+%22Myocardial+Ischemia%22%5BMesh%5D%29+OR+%22Myocardial+Infarction%22%5BMesh%5D%29+OR+%22Lung+Diseases%22%5BMesh%5D%29+OR+%22Respiration+Disorders%22%5BMesh%5D%29+OR+%22Heart+Diseases%22%5BMesh%5D%29+OR+%22Hemodynamics%22%5BMesh%5D) | 15:26:54 |

**EMBASE**

| \| Search history sorted by search number ascending \| \| \| \| \| \| \| \| --- \| --- \| --- \| --- \| --- \| --- \| --- \| \|  \| [# ▲](http://ovidsp.dc2.ovid.com.proxy.bib.uottawa.ca/ovid-b/ovidweb.cgi?&S=ECJPFPBCEJEBPAHKJPAKOEBFJLCAAA00&Sort+Sets=descending) \| **Searches** \| **Results** \| **Type** \| **Actions** \| **Annotations** \| \|  \| \| \| \| \| \| \| \|  \| 1 \| cerebrovascular accident/co, dm [Complication, Disease Management] \| 14983 \| Advanced \| [Display Results](http://ovidsp.dc2.ovid.com.proxy.bib.uottawa.ca/ovid-b/ovidweb.cgi?&S=ECJPFPBCEJEBPAHKJPAKOEBFJLCAAA00&SELECT=S.sh%7c&R=1&Process+Action=display)  [More](http://ovidsp.dc2.ovid.com.proxy.bib.uottawa.ca/ovid-b/ovidweb.cgi) \|  \| \|  \| 2 \| autonomic dysreflexia/ \| 1234 \| Advanced \| [Display Results](http://ovidsp.dc2.ovid.com.proxy.bib.uottawa.ca/ovid-b/ovidweb.cgi?&S=ECJPFPBCEJEBPAHKJPAKOEBFJLCAAA00&SELECT=S.sh%7c&R=2&Process+Action=display)  [More](http://ovidsp.dc2.ovid.com.proxy.bib.uottawa.ca/ovid-b/ovidweb.cgi) \|  \| \|  \| 3 \| heart infarction/ \| 271906 \| Advanced \| [Display Results](http://ovidsp.dc2.ovid.com.proxy.bib.uottawa.ca/ovid-b/ovidweb.cgi?&S=ECJPFPBCEJEBPAHKJPAKOEBFJLCAAA00&SELECT=S.sh%7c&R=3&Process+Action=display)  [More](http://ovidsp.dc2.ovid.com.proxy.bib.uottawa.ca/ovid-b/ovidweb.cgi) \|  \| \|  \| 4 \| heart muscle ischemia/ \| 94330 \| Advanced \| [Display Results](http://ovidsp.dc2.ovid.com.proxy.bib.uottawa.ca/ovid-b/ovidweb.cgi?&S=ECJPFPBCEJEBPAHKJPAKOEBFJLCAAA00&SELECT=S.sh%7c&R=4&Process+Action=display)  [More](http://ovidsp.dc2.ovid.com.proxy.bib.uottawa.ca/ovid-b/ovidweb.cgi) \|  \| \|  \| 5 \| lung injury/ \| 34624 \| Advanced \| [Display Results](http://ovidsp.dc2.ovid.com.proxy.bib.uottawa.ca/ovid-b/ovidweb.cgi?&S=ECJPFPBCEJEBPAHKJPAKOEBFJLCAAA00&SELECT=S.sh%7c&R=5&Process+Action=display)  [More](http://ovidsp.dc2.ovid.com.proxy.bib.uottawa.ca/ovid-b/ovidweb.cgi) \|  \| \|  \| 6 \| heart hemodynamics/ or cardiopulmonary hemodynamics/ or lung hemodynamics/ \| 30984 \| Advanced \| [Display Results](http://ovidsp.dc2.ovid.com.proxy.bib.uottawa.ca/ovid-b/ovidweb.cgi?&S=ECJPFPBCEJEBPAHKJPAKOEBFJLCAAA00&SELECT=S.sh%7c&R=6&Process+Action=display)  [More](http://ovidsp.dc2.ovid.com.proxy.bib.uottawa.ca/ovid-b/ovidweb.cgi) \|  \| \|  \| 7 \| 1 or 2 or 3 or 4 or 5 or 6 \| 421636 \| Advanced \| [Display Results](http://ovidsp.dc2.ovid.com.proxy.bib.uottawa.ca/ovid-b/ovidweb.cgi?&S=ECJPFPBCEJEBPAHKJPAKOEBFJLCAAA00&SELECT=S.sh%7c&R=7&Process+Action=display)  [More](http://ovidsp.dc2.ovid.com.proxy.bib.uottawa.ca/ovid-b/ovidweb.cgi) \|  \| \|  \| 8 \| anesthetic agent/ \| 43662 \| Advanced \| [Display Results](http://ovidsp.dc2.ovid.com.proxy.bib.uottawa.ca/ovid-b/ovidweb.cgi?&S=ECJPFPBCEJEBPAHKJPAKOEBFJLCAAA00&SELECT=S.sh%7c&R=8&Process+Action=display)  [More](http://ovidsp.dc2.ovid.com.proxy.bib.uottawa.ca/ovid-b/ovidweb.cgi) \|  \| \|  \| 9 \| analgesic agent/ \| 89159 \| Advanced \| [Display Results](http://ovidsp.dc2.ovid.com.proxy.bib.uottawa.ca/ovid-b/ovidweb.cgi?&S=ECJPFPBCEJEBPAHKJPAKOEBFJLCAAA00&SELECT=S.sh%7c&R=9&Process+Action=display)  [More](http://ovidsp.dc2.ovid.com.proxy.bib.uottawa.ca/ovid-b/ovidweb.cgi) \|  \| \|  \| 10 \| 8 or 9 \| 129093 \| Advanced \| [Display Results](http://ovidsp.dc2.ovid.com.proxy.bib.uottawa.ca/ovid-b/ovidweb.cgi?&S=ECJPFPBCEJEBPAHKJPAKOEBFJLCAAA00&SELECT=S.sh%7c&R=10&Process+Action=display)  [More](http://ovidsp.dc2.ovid.com.proxy.bib.uottawa.ca/ovid-b/ovidweb.cgi) \|  \| \|  \| 11 \| quadriplegia/ \| 17578 \| Advanced \| [Display Results](http://ovidsp.dc2.ovid.com.proxy.bib.uottawa.ca/ovid-b/ovidweb.cgi?&S=ECJPFPBCEJEBPAHKJPAKOEBFJLCAAA00&SELECT=S.sh%7c&R=11&Process+Action=display)  [More](http://ovidsp.dc2.ovid.com.proxy.bib.uottawa.ca/ovid-b/ovidweb.cgi) \|  \| \|  \| 12 \| autonomic dysreflexia.ab,ti. \| 1191 \| Advanced \| [Display Results](http://ovidsp.dc2.ovid.com.proxy.bib.uottawa.ca/ovid-b/ovidweb.cgi?&S=ECJPFPBCEJEBPAHKJPAKOEBFJLCAAA00&SELECT=S.sh%7c&R=12&Process+Action=display)  [More](http://ovidsp.dc2.ovid.com.proxy.bib.uottawa.ca/ovid-b/ovidweb.cgi) \|  \| \|  \| 13 \| myocardial infarction.ab,ti. \| 270115 \| Advanced \| [Display Results](http://ovidsp.dc2.ovid.com.proxy.bib.uottawa.ca/ovid-b/ovidweb.cgi?&S=ECJPFPBCEJEBPAHKJPAKOEBFJLCAAA00&SELECT=S.sh%7c&R=13&Process+Action=display)  [More](http://ovidsp.dc2.ovid.com.proxy.bib.uottawa.ca/ovid-b/ovidweb.cgi) \|  \| \|  \| 14 \| myocardial ischemia.ab,ti. \| 36540 \| Advanced \| [Display Results](http://ovidsp.dc2.ovid.com.proxy.bib.uottawa.ca/ovid-b/ovidweb.cgi?&S=ECJPFPBCEJEBPAHKJPAKOEBFJLCAAA00&SELECT=S.sh%7c&R=14&Process+Action=display)  [More](http://ovidsp.dc2.ovid.com.proxy.bib.uottawa.ca/ovid-b/ovidweb.cgi) \|  \| \|  \| 15 \| pulmonary deficit.ab,ti. \| 12 \| Advanced \| [Display Results](http://ovidsp.dc2.ovid.com.proxy.bib.uottawa.ca/ovid-b/ovidweb.cgi?&S=ECJPFPBCEJEBPAHKJPAKOEBFJLCAAA00&SELECT=S.sh%7c&R=15&Process+Action=display)  [More](http://ovidsp.dc2.ovid.com.proxy.bib.uottawa.ca/ovid-b/ovidweb.cgi) \|  \| \|  \| 16 \| pulmonary dysfunction.ab,ti. \| 3014 \| Advanced \| [Display Results](http://ovidsp.dc2.ovid.com.proxy.bib.uottawa.ca/ovid-b/ovidweb.cgi?&S=ECJPFPBCEJEBPAHKJPAKOEBFJLCAAA00&SELECT=S.sh%7c&R=16&Process+Action=display)  [More](http://ovidsp.dc2.ovid.com.proxy.bib.uottawa.ca/ovid-b/ovidweb.cgi) \|  \| \|  \| 17 \| respiratory acidosis.ab,ti. \| 2842 \| Advanced \| [Display Results](http://ovidsp.dc2.ovid.com.proxy.bib.uottawa.ca/ovid-b/ovidweb.cgi?&S=ECJPFPBCEJEBPAHKJPAKOEBFJLCAAA00&SELECT=S.sh%7c&R=17&Process+Action=display)  [More](http://ovidsp.dc2.ovid.com.proxy.bib.uottawa.ca/ovid-b/ovidweb.cgi) \|  \| \|  \| 18 \| respiratory alkalosis.ab,ti. \| 1395 \| Advanced \| [Display Results](http://ovidsp.dc2.ovid.com.proxy.bib.uottawa.ca/ovid-b/ovidweb.cgi?&S=ECJPFPBCEJEBPAHKJPAKOEBFJLCAAA00&SELECT=S.sh%7c&R=18&Process+Action=display)  [More](http://ovidsp.dc2.ovid.com.proxy.bib.uottawa.ca/ovid-b/ovidweb.cgi) \|  \| \|  \| 19 \| pulmonary complication.ab,ti. \| 1473 \| Advanced \| [Display Results](http://ovidsp.dc2.ovid.com.proxy.bib.uottawa.ca/ovid-b/ovidweb.cgi?&S=ECJPFPBCEJEBPAHKJPAKOEBFJLCAAA00&SELECT=S.sh%7c&R=19&Process+Action=display)  [More](http://ovidsp.dc2.ovid.com.proxy.bib.uottawa.ca/ovid-b/ovidweb.cgi) \|  \| \|  \| 20 \| complication.ab,ti. \| 440483 \| Advanced \| [Display Results](http://ovidsp.dc2.ovid.com.proxy.bib.uottawa.ca/ovid-b/ovidweb.cgi?&S=ECJPFPBCEJEBPAHKJPAKOEBFJLCAAA00&SELECT=S.sh%7c&R=20&Process+Action=display)  [More](http://ovidsp.dc2.ovid.com.proxy.bib.uottawa.ca/ovid-b/ovidweb.cgi) \|  \| \|  \| 21 \| 11 or 12 or 13 or 14 or 15 or 16 or 17 or 18 or 19 or 20 \| 752976 \| Advanced \| [Display Results](http://ovidsp.dc2.ovid.com.proxy.bib.uottawa.ca/ovid-b/ovidweb.cgi?&S=ECJPFPBCEJEBPAHKJPAKOEBFJLCAAA00&SELECT=S.sh%7c&R=21&Process+Action=display)  [More](http://ovidsp.dc2.ovid.com.proxy.bib.uottawa.ca/ovid-b/ovidweb.cgi) \|  \| \|  \| 22 \| "anesthetic*".ab,ti. \| 84629 \| Advanced \| [Display Results](http://ovidsp.dc2.ovid.com.proxy.bib.uottawa.ca/ovid-b/ovidweb.cgi?&S=ECJPFPBCEJEBPAHKJPAKOEBFJLCAAA00&SELECT=S.sh%7c&R=22&Process+Action=display)  [More](http://ovidsp.dc2.ovid.com.proxy.bib.uottawa.ca/ovid-b/ovidweb.cgi) \|  \| \|  \| 23 \| "anesthes*".ab,ti. \| 228593 \| Advanced \| [Display Results](http://ovidsp.dc2.ovid.com.proxy.bib.uottawa.ca/ovid-b/ovidweb.cgi?&S=ECJPFPBCEJEBPAHKJPAKOEBFJLCAAA00&SELECT=S.sh%7c&R=23&Process+Action=display)  [More](http://ovidsp.dc2.ovid.com.proxy.bib.uottawa.ca/ovid-b/ovidweb.cgi) \|  \| \|  \| 24 \| "sedativ*".ab,ti. \| 27925 \| Advanced \| [Display Results](http://ovidsp.dc2.ovid.com.proxy.bib.uottawa.ca/ovid-b/ovidweb.cgi?&S=ECJPFPBCEJEBPAHKJPAKOEBFJLCAAA00&SELECT=S.sh%7c&R=24&Process+Action=display)  [More](http://ovidsp.dc2.ovid.com.proxy.bib.uottawa.ca/ovid-b/ovidweb.cgi) \|  \| \|  \| 25 \| "anelges*".ab,ti. \| 7 \| Advanced \| [Display Results](http://ovidsp.dc2.ovid.com.proxy.bib.uottawa.ca/ovid-b/ovidweb.cgi?&S=ECJPFPBCEJEBPAHKJPAKOEBFJLCAAA00&SELECT=S.sh%7c&R=25&Process+Action=display)  [More](http://ovidsp.dc2.ovid.com.proxy.bib.uottawa.ca/ovid-b/ovidweb.cgi) \|  \| \|  \| 26 \| 22 or 23 or 24 or 25 \| 297056 \| Advanced \| [Display Results](http://ovidsp.dc2.ovid.com.proxy.bib.uottawa.ca/ovid-b/ovidweb.cgi?&S=ECJPFPBCEJEBPAHKJPAKOEBFJLCAAA00&SELECT=S.sh%7c&R=26&Process+Action=display)  [More](http://ovidsp.dc2.ovid.com.proxy.bib.uottawa.ca/ovid-b/ovidweb.cgi) \|  \| \|  \| 27 \| "quadripleg*".ab,ti. \| 5489 \| Advanced \| [Display Results](http://ovidsp.dc2.ovid.com.proxy.bib.uottawa.ca/ovid-b/ovidweb.cgi?&S=ECJPFPBCEJEBPAHKJPAKOEBFJLCAAA00&SELECT=S.sh%7c&R=27&Process+Action=display)  [More](http://ovidsp.dc2.ovid.com.proxy.bib.uottawa.ca/ovid-b/ovidweb.cgi) \|  \| \|  \| 28 \| "tetrapleg*".ab,ti. \| 5763 \| Advanced \| [Display Results](http://ovidsp.dc2.ovid.com.proxy.bib.uottawa.ca/ovid-b/ovidweb.cgi?&S=ECJPFPBCEJEBPAHKJPAKOEBFJLCAAA00&SELECT=S.sh%7c&R=28&Process+Action=display)  [More](http://ovidsp.dc2.ovid.com.proxy.bib.uottawa.ca/ovid-b/ovidweb.cgi) \|  \| \|  \| 29 \| 27 or 28 \| 11138 \| Advanced \| [Display Results](http://ovidsp.dc2.ovid.com.proxy.bib.uottawa.ca/ovid-b/ovidweb.cgi?&S=ECJPFPBCEJEBPAHKJPAKOEBFJLCAAA00&SELECT=S.sh%7c&R=29&Process+Action=display)  [More](http://ovidsp.dc2.ovid.com.proxy.bib.uottawa.ca/ovid-b/ovidweb.cgi) \|  \| \|  \| 30 \| 7 or 21 \| 983503 \| Advanced \| [Display Results](http://ovidsp.dc2.ovid.com.proxy.bib.uottawa.ca/ovid-b/ovidweb.cgi?&S=ECJPFPBCEJEBPAHKJPAKOEBFJLCAAA00&SELECT=S.sh%7c&R=30&Process+Action=display)  [More](http://ovidsp.dc2.ovid.com.proxy.bib.uottawa.ca/ovid-b/ovidweb.cgi) \|  \| \|  \| 31 \| 10 or 26 \| 399488 \| Advanced \| [Display Results](http://ovidsp.dc2.ovid.com.proxy.bib.uottawa.ca/ovid-b/ovidweb.cgi?&S=ECJPFPBCEJEBPAHKJPAKOEBFJLCAAA00&SELECT=S.sh%7c&R=31&Process+Action=display)  [More](http://ovidsp.dc2.ovid.com.proxy.bib.uottawa.ca/ovid-b/ovidweb.cgi) \|  \| \|  \| 32 \| 11 or 29 \| 20601 \| Advanced \| [Display Results](http://ovidsp.dc2.ovid.com.proxy.bib.uottawa.ca/ovid-b/ovidweb.cgi?&S=ECJPFPBCEJEBPAHKJPAKOEBFJLCAAA00&SELECT=S.sh%7c&R=32&Process+Action=display)  [More](http://ovidsp.dc2.ovid.com.proxy.bib.uottawa.ca/ovid-b/ovidweb.cgi) \|  \| \|  \| 33 \| 30 and 31 and 32 \| 376 \| Advanced \| [Display Results](http://ovidsp.dc2.ovid.com.proxy.bib.uottawa.ca/ovid-b/ovidweb.cgi?&S=ECJPFPBCEJEBPAHKJPAKOEBFJLCAAA00&SELECT=S.sh%7c&R=33&Process+Action=display)  [More](http://ovidsp.dc2.ovid.com.proxy.bib.uottawa.ca/ovid-b/ovidweb.cgi) \|  \| \| Combine with:  \| \| \|  \|  \| \| \| | [Contract](http://ovidsp.dc2.ovid.com.proxy.bib.uottawa.ca/ovid-b/ovidweb.cgi?&S=ECJPFPBCEJEBPAHKJPAKOEBFJLCAAA00&SELECT=S.sh%7c&Contract=1&Main+Search+Page=Main+Search+Page) |
| --- | --- | --- | --- | --- | --- | --- | --- | --- | --- | --- | --- | --- | --- | --- | --- | --- | --- | --- | --- | --- | --- | --- | --- | --- | --- | --- | --- | --- | --- | --- | --- | --- | --- | --- | --- | --- | --- | --- | --- | --- | --- | --- | --- | --- | --- | --- | --- | --- | --- | --- | --- | --- | --- | --- | --- | --- | --- | --- | --- | --- | --- | --- | --- | --- | --- | --- | --- | --- | --- | --- | --- | --- | --- | --- | --- | --- | --- | --- | --- | --- | --- | --- | --- | --- | --- | --- | --- | --- | --- | --- | --- | --- | --- | --- | --- | --- | --- | --- | --- | --- | --- | --- | --- | --- | --- | --- | --- | --- | --- | --- | --- | --- | --- | --- | --- | --- | --- | --- | --- | --- | --- | --- | --- | --- | --- | --- | --- | --- | --- | --- | --- | --- | --- | --- | --- | --- | --- | --- | --- | --- | --- | --- | --- | --- | --- | --- | --- | --- | --- | --- | --- | --- | --- | --- | --- | --- | --- | --- | --- | --- | --- | --- | --- | --- | --- | --- | --- | --- | --- | --- | --- | --- | --- | --- | --- | --- | --- | --- | --- | --- | --- | --- | --- | --- | --- | --- | --- | --- | --- | --- | --- | --- | --- | --- | --- | --- | --- | --- | --- | --- | --- | --- | --- | --- | --- | --- | --- | --- | --- | --- | --- | --- | --- | --- | --- | --- | --- | --- | --- | --- | --- | --- | --- | --- | --- | --- | --- | --- | --- | --- | --- | --- | --- | --- | --- | --- | --- | --- | --- | --- | --- | --- | --- | --- | --- | --- | --- | --- | --- | --- | --- | --- | --- | --- | --- | --- | --- | --- | --- | --- |
